# Context-Specificity of Locomotor Learning Is Developed during Childhood

**DOI:** 10.1523/ENEURO.0369-21.2022

**Published:** 2022-04-18

**Authors:** Dulce M. Mariscal, Erin V. L. Vasudevan, Laura A. Malone, Gelsy Torres-Oviedo, Amy J. Bastian

**Affiliations:** 1 Bioengineering Department, University of Pittsburgh, Pittsburgh, PA 15260; 2 Center for Neural Basis of Cognition, University of Pittsburgh, Pittsburgh, PA 15213; 3 Neuroscience Department, Johns Hopkins University, Baltimore, MD, 21205; 4 Neurology Department, Johns Hopkins University, Baltimore, MD, 21205; 5 Physical Medicine, and Rehabilitation Department, Johns Hopkins University, Baltimore, MD, 21205; 6 Kennedy Krieger Institute, Baltimore, MD, 21205; 7 School of Health Technology and Management, Stony Brook University, Stony Brook, NY, 11794

**Keywords:** development, generalization, kinematics, locomotion, motor adaptation, motor control

## Abstract

Humans can perform complex movements with speed and agility in the face of constantly changing task demands. To accomplish this, motor plans are adapted to account for errors in our movements because of changes in our body (e.g., growth or injury) or in the environment (e.g., walking on sand vs ice). It has been suggested that adaptation that occurs in response to changes in the state of our body will generalize across different movement contexts and environments, whereas adaptation that occurs with alterations in the external environment will be context-specific. Here, we asked whether the ability to form generalizable versus context-specific motor memories develops during childhood. We performed a cross-sectional study of context-specific locomotor adaptation in 35 children (3–18 years old) and 7 adults (19–31 years old). Subjects first adapted their gait and learned a new walking pattern on a split-belt treadmill, which has two belts that move each leg at a different speed. Then, subjects walked overground to assess the generalization of the adapted walking pattern across different environments. Our results show that the generalization of treadmill after-effects to overground walking decreases as subjects’ age increases, indicating that age and experience are critical factors regulating the specificity of motor learning. Our results suggest that although basic locomotor patterns are established by two years of age, brain networks required for context-specific locomotor learning are still being developed throughout youth.

## Significance Statement

The generalization of motor learning from experienced situations to new ones is important for devising optimal rehabilitation programs. Little is known about how the motor system develops the ability to form generalizable versus context-specific motor memories. Here, we find that younger children generalize locomotor patterns to a much greater extent than older children and adults. These results are significant because they demonstrate that the ability to form context-specific locomotor memories develops during childhood.

## Introduction

As we interact with the world, we learn how to shape our movements to optimally perform a task in the situation at hand. One form of motor learning is often referred to as adaptation and is driven by movement errors. Through practice, one learns to reduce errors and restore a more optimal movement pattern. When the perturbation is removed, people gradually unlearn the adapted movement. It is known that the mature motor system can generalize this type of learning to untrained movements within the same environment. For example, we can learn to counteract predictable visual or force disturbances during reaching and show some generalization to untrained reaches using the other arm (Sainburg and Wang, 2002; Criscimagna-Hemminger et al., 2003; Malfait and Ostry, 2004) or reaching in new directions (Krakauer et al., 2000; Thoroughman and Shadmehr, 2000; Donchin et al., 2003; Day et al., 2016). Generalization is not robust when moving outside of the training environment. This type of learning does not necessarily influence the exact same movement performed in other environments (Cothros et al., 2006; Kluzik et al., 2008). For example, adults adapt their walking on a split-belt treadmill that moves their legs at different speeds but switch back to their original gait (i.e., movements before split-belt adaptation) after a few steps of walking overground (Reisman et al., 2009; Torres-Oviedo and Bastian, 2010, 2012). This motor specificity is possible given contextual information specific to the training situation provided by sensory cues (Krouchev and Kalaska, 2003; Wada et al., 2003; Osu et al., 2004; Ahmed et al., 2008; Cothros et al., 2009; Ingram et al., 2010; Addou et al., 2011; Hirashima and Nozaki, 2012; Howard et al., 2013) or body-state cues (Gandolfo et al., 1996; Langan and Seidler, 2011; Howard et al., 2012, 2013, 2015; Torres-Oviedo and Bastian, 2012). Thus, contextual information about the environment as we acquire new motor patterns contributes to the context-specificity of encoded movements.

The specificity of movements across environments may also depend heavily on the ability to switch actions according to the context. Several studies have shown that healthy aging has an impact on the motor system’s ability to transition between different context-specific motor memories (Bock, 2005; Bock and Girgenrath, 2006; Wang et al., 2011; Sombric et al., 2017; Sombric and Torres-Oviedo, 2021). For instance, older adults have greater difficulties switching between the split-belt and the regular walking patterns compared with young adults (Sombric et al., 2017; Sombric and Torres-Oviedo, 2021). Therefore, the generalization from one environment to another can be the by-product of slow switching (i.e., difficulty switching between locomotor patterns on environmental transitions). The ability to rapidly switch actions might require interaction with different environments and thus, it might be developed throughout childhood.

There are few studies of generalization in young children compared with older children. In these studies, children were tested for generalization of a trained behavior to untrained movements within the same environment. Studies of a learned arm movement show reduced or absent generalization to the other arm in tasks like the Purdue Pegboard Test (De Almeida Batista et al., 2017), target pursuit (Byrd et al., 1986), visuomotor actions (Uehara, 1998), or throwing (Sidaway et al., 2012). In infants, environmental demands learned when crawling do not transfer to walking when performed in exactly the same environment (Kretch and Adolph, 2013). These studies suggest that young children link the learned behavior to the trained effector or action and do not generalize their learning to other movements within the same environment. This type of learning would lead young children to show greater generalization of a motor pattern adapted in one environment (e.g., split-belt treadmill) to a different environment (e.g., overground) compared with older children or adults. A recent study, however, has shown that children can form context-specific memories of the split-belt treadmill that are not degraded by performing the same movement in a different context (Musselman et al., 2016), raising the possibility that age does not impact generalization of walking across different environments. In this work, we explicitly addressed the question of whether human development altered the generalization of locomotor learning.

To test the effect of human development on the generalization of movements, we performed a cross-sectional study in which children walked on a split-belt treadmill or overground after acquiring a new walking pattern on the treadmill. Note that contrary to other developmental studies, we tested the same motor behavior (i.e., children walked in both situations) across environments that had different dynamics (i.e., split-belt treadmill vs overground). We chose to test the generalization of the split-belt learning because it has been shown to develop robust context-specific motor memories in adults (Reisman et al., 2009; Torres-Oviedo and Bastian, 2012) and children can acquire the split-belt walking pattern as young as two years old when they are proficient walkers (Vasudevan et al., 2011; Patrick et al., 2014).

## Materials and Methods

### Subjects

Thirty-five children (24 males and 11 females; aged 3.1–17.9 years; mean ± SD, 10.6 ± 4.2 years) and seven young adults (four males and three females; aged 19.6–31.5 years; mean ± SD, 25.5 ± 4.0 years) participated in this study. These subjects were divided into six age groups: 3–5, 6–8, 9–11, 12–14, 15–17 years old (y/o), and adults (>18 y/o). Characteristics of each group are shown in Table 1. The experimental protocol was approved by the Institutional Review Board at the Johns Hopkins University, and all subjects and/or their parent or legal guardian gave informed written consent before participating in the study.

**Table 1.** Age group characteristics

Age group	Number of subjects	Number of females	Age range (years)	Mean ± SD (years)	Mean speeds (m/s; slow, fast)
3–5 y/o	7	2	3.1**–**5.9	4.7 ± 0.9	(0.49, 0.98)
6–8 y/o	7	2	6.5**–**9.0	7.9 ± 1.0	(0.79, 1.57)
9–11 y/o	7	2	9.4**–**11.0	10.5 ± 0.6	(0.75, 1.5)
12–14 y/o	7	2	12.4**–**14.3	13.3 ± 0.7	(0.88, 1.76)
15–17 y/o	7	3	15.1**–**17.9	16.3 ± 1.2	(0.95, 1.90)
Young adults (>18 y/o)	7	3	19.6**–**31.5	25.5 ± 4.0	(0.94, 1.89)

### Experimental setup and design

Participants adapted their walking pattern on a split-belt treadmill and we tested the transfer of this learning to overground walking (i.e., off of the treadmill). Locomotor adaptation was achieved using a split-belt treadmill (Woodway USA) that drove the speed of each leg independently. This paradigm has been demonstrated to induce the storage of a modified walking pattern that is expressed as an aftereffect in “tied-belt” walking (i.e., legs moving at the same speed), and must be de-adapted to return to normal walking (Reisman et al., 2005). Participants were positioned in the middle of the treadmill, with one foot on each belt. A thin wooden panel was placed in between the two belts to ensure subjects would not step on the opposite belt when belts were moving at different speeds (i.e., split-belt condition). Participants of all ages held onto a front handrail adjusted to elbow height and wore a safety harness that did not support their weight during walking. All subjects watched TV while walking.

**Figure 1.**
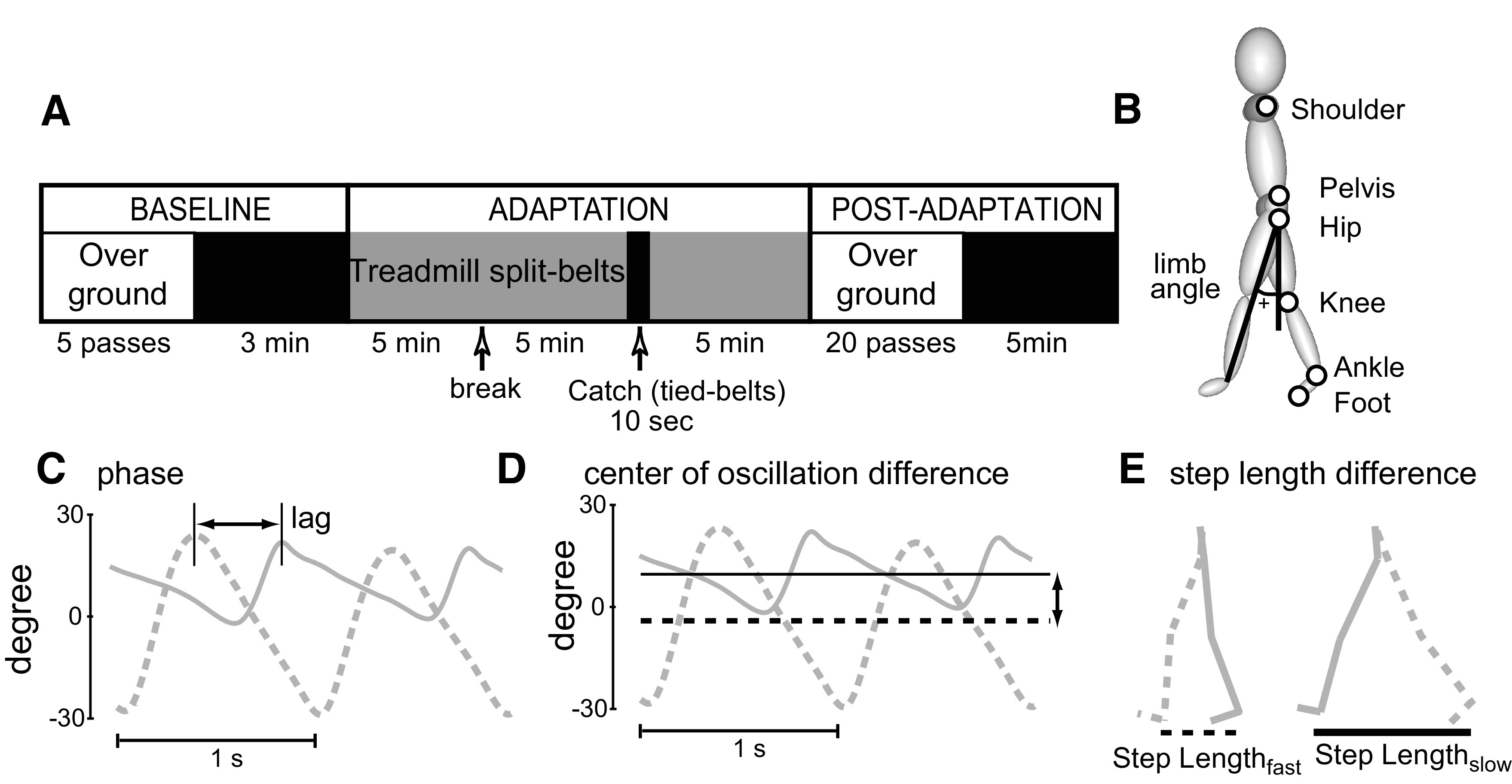
***A***, Overall paradigm. In all groups, baseline behavior was recorded overground and subsequently on the treadmill. Then subjects were adapted for a total of 15 min divided into three blocks of 5 min each separated by a break. A 10-s catch condition was introduced when subjects had been adapted for 10 min. Aftereffects were also assessed overground after 5 min of re-adaptation to the split condition. Finally, subjects returned to the treadmill where they walked for 5 min to determine the washout of learning specific to the treadmill from the remaining aftereffects. ***B***, Diagram of marker locations. Limb angle convention is shown on the stick figure. ***C***, Limb angle trajectories plotted as a function of time in early split-belt adaptation; two cycles are shown. Limb angles are positive when the limb is in front of the trunk (flexion). Phase quantifies the lag producing the largest cross-correlation between the two legs. When the legs move in anti-phase, the lag of 0.5 leads to the largest cross-correlation. ***D***, Limb angle trajectories are shown in gray and angle axes about which each leg oscillates are shown in black. In this example, slow limb (solid line) oscillates more forward with respect to the vertical axis than the fast limb (dashed line). The center of oscillation quantifies the difference in where the legs oscillate, illustrated by the distance between two black lines. ***E***, An example of kinematic data of two consecutive steps is shown. Kinematic data for every two steps were used to calculate step symmetry, defined as the difference in step lengths normalized by the sum of step lengths.*Figure Contributions*: Gelsy Torres-Oviedo created schematics for general paradigm and gait parameters.

We recorded the motor behavior of all participants during baseline, adaptation, and postadaptation periods. Baseline and postadaptation were collected off and on the treadmill (Fig. 1*A*). We collected a baseline period before adaptation in which subjects walked with the belts moving together (i.e., “tied-belt” walking) at three different speeds: a slow, a fast, and an intermediate speed for 1 min each. Specifically, the slow speed was set equal to the participant’s leg length in m/s, as done before (Vasudevan et al., 2011). Leg length was defined as the distance from the lateral malleolus to the iliac crest (i.e., in Fig. 1*B*, ankle and pelvis marker, respectively). Consequently, the slow speed ranged between 0.4 and 0.98 m/s. The fast speed was equal to two times the slow speed and the intermediate speed was an average of the slow and fast speeds. We scaled walking speeds with this method to ensure subjects from all ages were perturbed similarly during the split-belt condition, when each belt was moving at the calculated slow and fast speeds (Vasudevan et al., 2011).

After baseline walking, subjects walked on the split-belt treadmill, where one belt moved at the slow speed and the other moved at the fast speed, for 15 min. During split-belt adaptation, we stopped the belts every 5 min to allow subjects to rest for ∼1 min. After the first two 5-min split-belt treadmill blocks, we collected a 10-s “catch” condition during which both belts were moving together at the slow speed (i.e., slow, tied-belt walking). The recordings during this “catch” condition allowed us to assess storage of the adaptation effects (i.e., aftereffects) on the treadmill. Subjects then walked in the split-belt condition for the final 5 min to re-adapt the walking pattern. The treadmill was stopped and re-started again at every speed transition. After the entire adaptation period, subjects were transported on a wheelchair from the treadmill to a 6-m walkway, to prevent them from taking any steps outside the motion capture area. Subjects walked on the walkway for 20 back-and-forth passes to test for transfer to overground walking of aftereffects because of the split-belt treadmill adaptation. Subjects were asked to avoid stepping outside of the motion capture area so that we could record as many of their initial steps after split-belt treadmill adaptation as possible. The self-selected walking speeds in all subjects ranged between 0.35 and 1.1 m/s. After assessing overground transfer, subjects returned to the treadmill and walked for 5 min in the tied-belts condition at the computed slow speed. This last period allowed us to test for washout of the treadmill aftereffects because of overground walking. We chose to assess aftereffects on the treadmill during catch and washout periods at the slow belt speed since it has been shown to induce the largest aftereffects (Vasudevan and Bastian, 2010). We allowed participants to walk at their preferred overground walking speed since aftereffects during overground walking are minimally affected by speed (Hamzey et al., 2016).

### Data collection

During all treadmill and overground walking, kinematic data were collected at 100 Hz using Optotrak (Northern Digital). Infrared-emitting markers were placed bilaterally over the following anatomic landmarks: foot (fifth metatarsal head), ankle (lateral malleolus), knee (lateral femoral epicondyle), hip (greater trochanter), pelvis (iliac crest), and shoulder (acromion process). Marker location is indicated in Figure 1*B*. The times of heel strike and toe-off (i.e., when the foot contacts and lifts off the ground) were recorded by foot-switches placed on the bottom of the shoes or were estimated from the ankle kinematic data. To avoid occlusion of the hip and pelvis markers during overground and treadmill walking because of arm-swing, participants walked with their arms crossed on the treadmill and overground, except for young children (less than six years old) who were unable to consistently maintain their arms crossed while walking. Thus, young children were instructed to walk walking holding a rail with both hand on the treadmill and overground while pushing a light (seven-pound) toy-shopping cart with adjustable rail height. All marker data were rotated to a reference frame centered on the subject’s body with the fore-aft axis aligned to the individual’s walking direction. This was done because younger children did not walk in a straight path during overground walking, which affected the step lengths’ calculation when using the laboratory reference frame. Thus, we estimated each subject’s walking direction in every stride as the perpendicular axis to the participant’s frontal plane, which was computed using the hip and pelvis markers. We subsequently rotated the fore-aft axis to be aligned to this estimated walking direction. We used the same approach for all individuals and all strides overground and on the treadmill for consistency purposes.

### Data analysis

#### Gait parameters

Temporal and spatial characteristics of gait that are known to adapt during split-belt treadmill walking were assessed (Malone and Bastian, 2010; Vasudevan et al., 2011). We specifically chose to characterize the generalization of gait parameters such as phase shift, center of oscillation, and step asymmetry to compare to prior generalization studies (Torres-Oviedo and Bastian, 2010, 2012) and developmental studies in split-belt walking (Vasudevan et al., 2011; Musselman et al., 2016). To quantify temporal gait features we used phase shift between the two legs. To this end, we computed the cross-correlation between limb angle trajectories during one full step cycle for each leg. Limb angle was defined as the angle between the vertical axis and the vector from hip to ankle on the *x-y* plane (Fig. 1*B*). Phase shift was the lag or lead-time for a maximum correlation between limb angle trajectories (Fig. 1*C*). A phase shift value of 0.5 would indicate that legs are moving in anti-phase. To correct for subjects’ biases, we subtracted the phase shift during the baseline period from all other periods. Consequently, a reported value of 0 indicates that legs are moving in anti-phase, positive phase shifts indicate that the fast leg is phase advanced relative to the slow leg, and negative phase shifts indicate that the fast leg is lagging the slow leg.

To quantify spatial gait features we used center of oscillation difference, which is defined as the difference between angles of oscillation of each leg (Fig. 1*D*). The angle of oscillation is defined as the angle between the vertical axis (0° axis in Fig. 1*D*) and the axis about which the leg is oscillating, illustrated by the black dashed and solid lines in Figure 1*D*. A center of oscillation value of 0 would indicate that both legs are oscillating about the same axis, a positive value would indicate that the leg on the fast belt is oscillating about an axis that is forward to the one of the leg on the slow belt, and a negative value would indicate that the leg on the slow belt is oscillating about an axis that is forward to the one of the leg on the fast belt (Vasudevan et al., 2011). We subtracted the center of oscillation values during the baseline period from all other periods to correct for subjects’ biases.

Finally, we also quantified step symmetry which is defined as the difference between step lengths of the two legs, where step length is the distance between two ankle markers at the time of heel strike of the leading leg (Fig. 1*E*). This difference was normalized by the step lengths’ sum to account for variations in step length across subjects of different leg lengths. A step length symmetry value of 0 would indicate that step lengths are equal, a positive value would indicate that the leg on the fast belt is taking longer steps, and a negative value would indicate that the leg on the slow belt is taking longer steps. Baseline subjects’ biases were removed by subtracting baseline values from all other experimental periods.

#### Outcome measures

We characterized the subjects’ adaptation and postadaptation behavior on the treadmill and overground with six outcome measures corresponding to different time-periods of the experiment: (1) adaptation before overground (Adapt; the difference between the average of first 5 and last 20 strides of the adaptation period); (2) aftereffects on the treadmill during the catch (TM_catch_; first five strides of catch condition; (3) early overground aftereffects (OG_early_; first five strides of overground postadaptation); (4) late overground aftereffects (OG_late_; last 20 strides of overground postadaptation); (5) early treadmill aftereffects (TM_early_; first five strides of treadmill postadaptation and after overground walking); and (6) late treadmill aftereffects (TM_late_; last 20 strides of treadmill postadaptation and after overground walking). Note that we quantified aftereffects at the end of the postadaptation periods to determine whether subjects were fully washed out at the end of each postadaptation period.

### Statistical analysis

We did a correlation analysis between the participants’ age and each of our six outcome measures**:** Adapt, TM_catch_, OG_early_, OG_late_, TM_early_, and TM_late_. The correlation analysis between Age and Adapt, or TM_catch_, respectively, was done to determine whether age affected the extent of adaptation, as quantified by the change in gait parameters during adaptation and by the size of the treadmill aftereffects during the catch condition. Similarly, the correlation analysis between Age and OG_early_ or OG_late_ was done to identify age-related changes on the carryover of recalibrated movements from the treadmill (training context) to overground (testing context). Moreover, the correlation analysis between age and TM_early_ or TM_late_ was done to determine whether age influenced the washout of aftereffects on the treadmill by overground walking. Finally, we evaluated whether the variability in gait asymmetry during baseline was a predictor of the aftereffects overground. This was done with separate correlation between OG_early_ versus baseline variability for each parameter (i.e., phase shift, center of oscillation, or step symmetry). Variability was calculated as the variance during treadmill baseline walking for each measure. We used the steps during treadmill baseline, rather than overground baseline, to have a more robust characterization of each participant’s variability given the relatively larger number of steps recorded during treadmill walking compared with overground walking. We also determined the association between age and step variability since these factors could be correlated.

In all correlation analyses, we used a Spearman’s rank-order correlation. We used a non-parametric correlation because after testing for normality our outcome measurements fail to pass the Kolmogorov–Smirnov test, *p* < 0.05 as a measure of significance in all correlation.

### Power analysis

The primary focus of this study was to test the hypothesis that age influences the generalization of movements from the treadmill to overground. A power analysis based on our previously published data (Sombric et al., 2017; Sombric and Torres-Oviedo, 2021) indicated that an estimated sample size of *n* = 11 would identify a one-sample side correlation coefficient of *r* = 0.71 with a power of 0.8. Thus, *n* = 35 would be sufficient to identify a significant correlation between age and generalization.

## Results

### All children adapted their gait during split-belt walking

**Figure 2.**
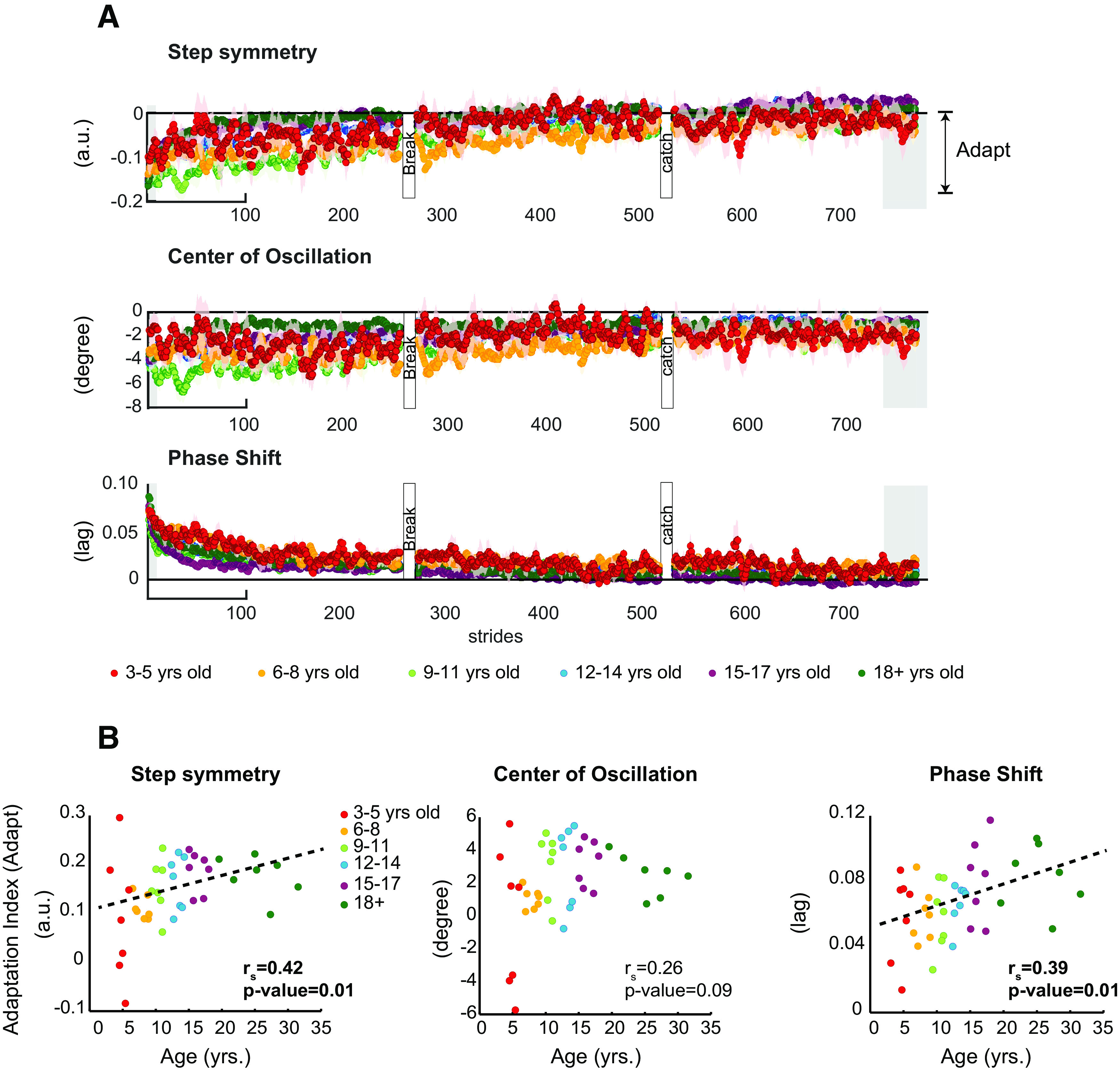
***A***, Time courses for gait parameters characterizing the behavior during split-belt walking of all age groups. Every dot represents the group average of five strides and color shaded areas indicate standard error (SE). Children adapted their gait more slowly during the split-belt condition. For visualization purposes, gray background indicates the strides used for computing Adapt, used in the scatter plots in panel ***B***. ***B***, Correlation results of Age versus Adapt before overground walking. Adapt quantified the extent to which subjects changed their gait during the adaptation period. Large numbers indicate more adjustments during split-belt walking. Dots indicate individual subjects’ data and colors indicate subjects age groups. We found a significant effect of age in phase shift and step length asymmetry, indicating that younger subjects adapted less over the same period of split-belt walking than older children or young adults. This effect is not observed in the center of oscillation, which is a more variable parameter. Dotted line is shown as reference for a linear regression. Arbitrary units (a.u.).*Figure Contributions*: Gelsy Torres-Oviedo, Erin Vasudevan, and Laura Malone performed the experiments. Dulce Mariscal and Gelsy Torres-Oviedo analyzed the data.

We found that participants of all ages adapted their gait on the split-belt treadmill; however, younger children did so more slowly. Figure 2*A* shows time courses during the adaptation period (split-belt condition) for all age groups. We observed that younger children adapted slower than adults as reported before (Vasudevan et al., 2011). This age-mediated effect is, for example, qualitatively shown by the slower adaptation of 9–11 years old (light green trace) compared with +18 years old (dark green trace). Consistently, younger subjects adapted less over the same period of split-belt walking than young adults. This is indicated by a significant positive association between age and the extent to which participants adjust their gait before overground walking (Adapt) for phase shift (Adapt: $r_{s}=0.389,p\;=\;0.014$) and step length asymmetry (Adapt: $r_{S}=0.43,p\;=\;0.005$; Fig. 2*B*). On the other hand, we did not observe an age effect in the adaptation of the center of oscillation (Adapt: $r_{s}=0.26,p=0.10$). Of note, we observed negative Adapt indices in a couple of youngest participants because their large step-to-step variability in spatial parameters (step length asymmetry and center of oscillation) made it difficult to quantify their initial adaptation values. Thus, these negative indices represent large step-to-step variability in these youngest subjects, rather than an increment in asymmetric stepping during the adaptation period. In other words, subjects from all ages were able to adapt their gait, but younger individuals did so to a lesser extent before we measured their aftereffects overground.

### While all subjects recalibrated their gait similarly, younger children transfer their adapted movements more

**Figure 3.**
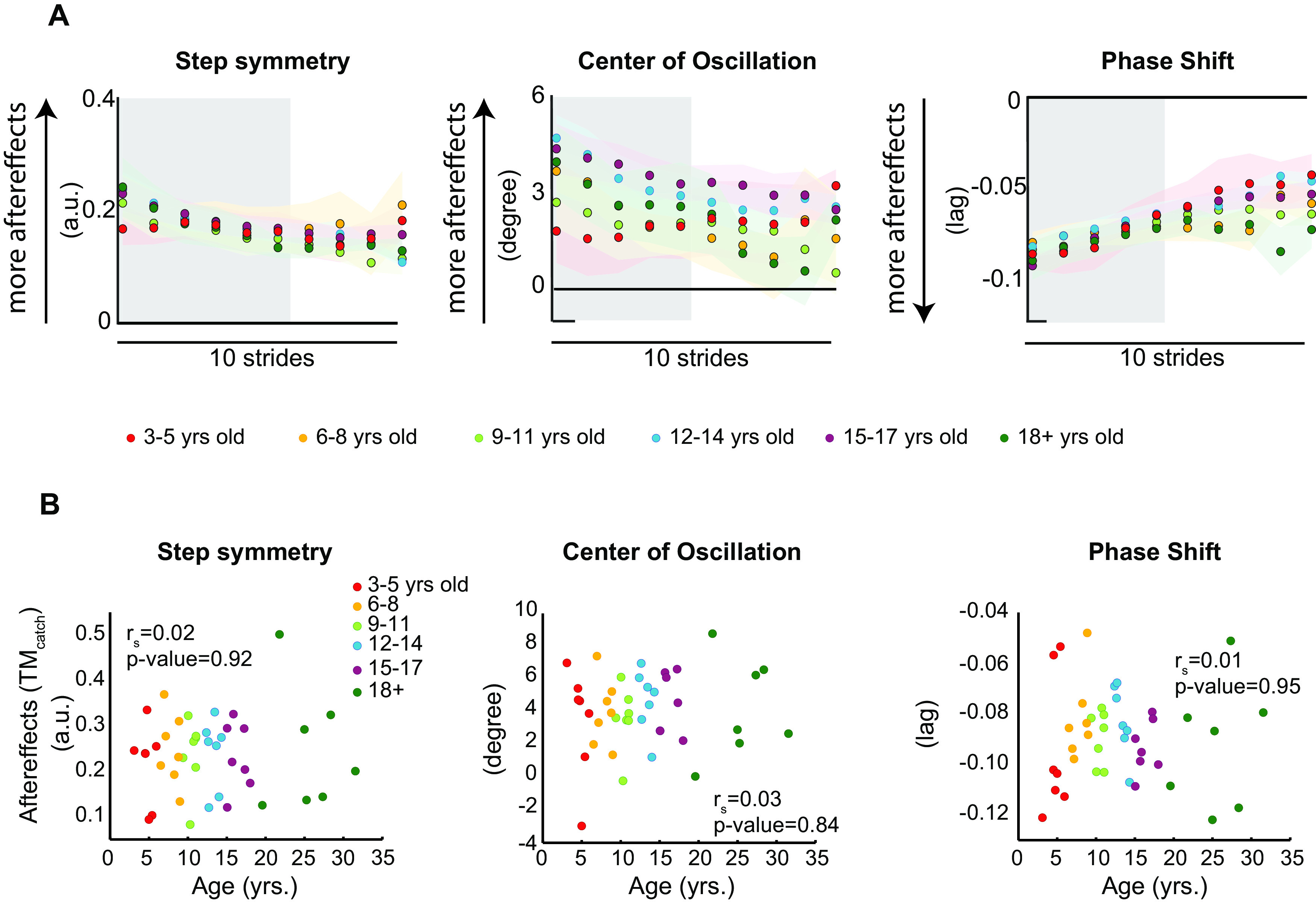
***A***, Time courses during catch condition for two age groups. Every dot represents the group average of five strides and color shaded areas indicate SE. Gray area indicates the strides used for the regression analysis displayed in panel ***B***. ***B***, Correlations results of Age versus aftereffects on the treadmill during the catch trial (TMcatch); *y*-axis indicates the magnitude of aftereffects on the treadmill TM*_catch_* for each individual; *x*-axis indicates the age of each participant at the time of the experiment. Dots indicate individual subjects’ data and colors indicate subjects’ age group. We did not find an age effect indicating that all individuals recalibrated their movements after split-belt walking. Arbitrary units (a.u.).*Figure Contributions*: Gelsy Torres-Oviedo, Erin Vasudevan, and Laura Malone performed the experiments. Dulce Mariscal and Gelsy Torres-Oviedo analyzed the data.

Age did not modulate the aftereffects on the treadmill (TM_catch_) after split-belt walking. This indicates that all subjects, regardless of their age, were able to recalibrate their locomotor pattern on the treadmill. Figure 3*A* shows the time courses of the treadmill aftereffects during the catch for all age groups. Note that all the curves overlap. Accordingly, we found that age was not a strong predictor of individuals’ TM_catch_ values for step symmetry ( $r_{s}=0.02,p=0.92$), center of oscillation ( $r_{s}=0.03,p=0.84$), or phase shift ( $r_{s}=0.010,p=0.95$; Fig. 3*B*). Recall that TM_catch_ quantifies the magnitude of aftereffects during the catch condition, and it is used to assess sensorimotor recalibration in the training context. In sum, kids were slower to adapt their gait, but they all experienced similar sensorimotor recalibration, as shown by the similar aftereffects during the catch condition after split-belt walking.

**Figure 4.**
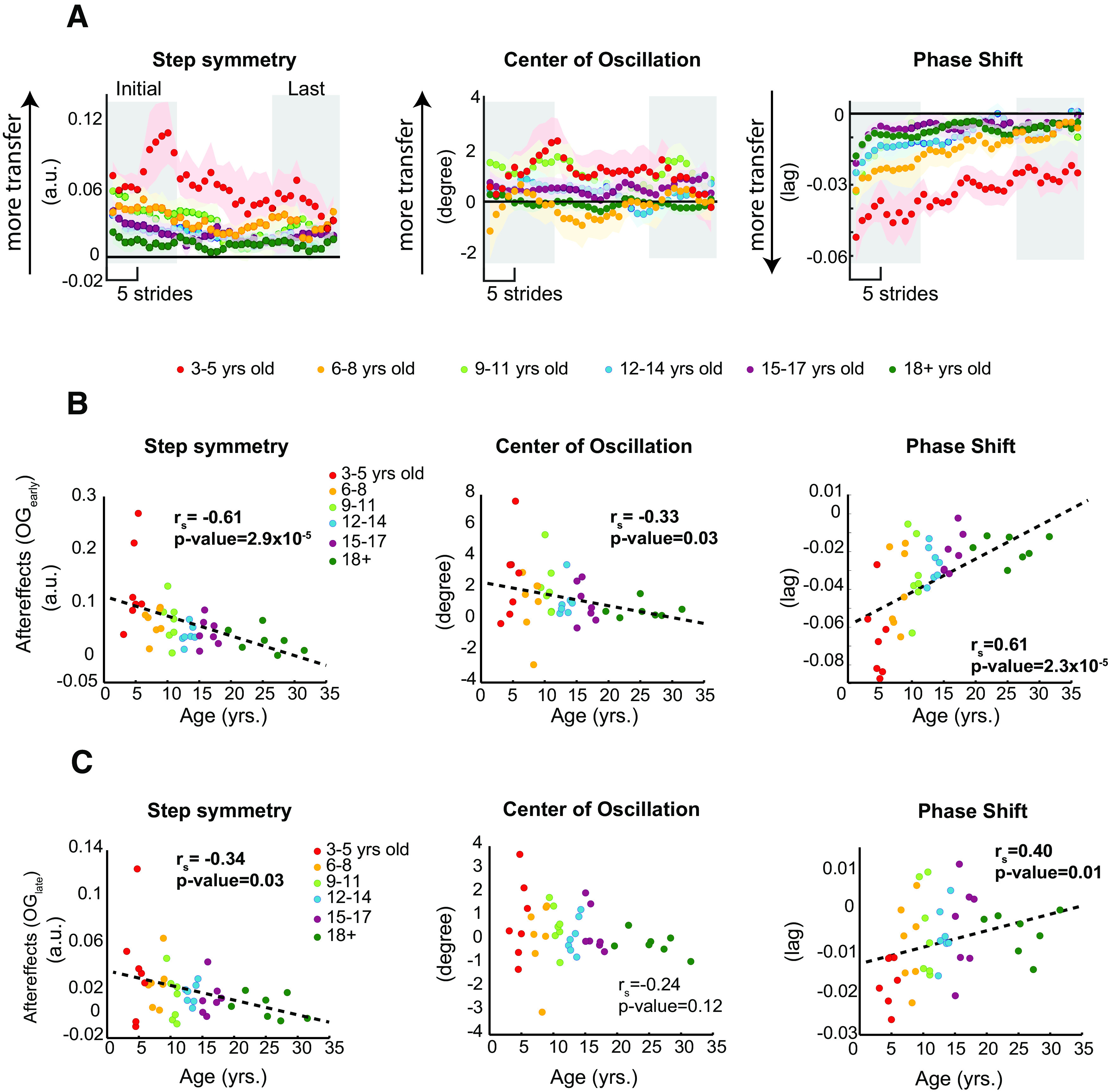
***A***, Time courses during over ground walking for two age groups. Every dot represents the group average of five strides and color shaded areas indicate SE. Gray area indicates the strides used for the regression analysis displayed in panels ***B***, ***C***. ***B***, ***C***, Regression analyses showing the relation between age and aftereffects during the initial OG*_early_* (panel ***B***) and the last OG*_late_* (panel ***C***) steps of the postadaptation period overground in all kinematic parameters. Dots indicate individual subjects’ data and colors indicate the different age groups. Age was a significant predictor of OG*_early_* and OG*_late_* in all step length asymmetry and phase shift, such that younger subjects exhibited greater aftereffects overground than older children and young adults. Dotted line is shown as reference for a linear regression. Arbitrary units (a.u.).*Figure Contributions*: Gelsy Torres-Oviedo, Erin Vasudevan, and Laura Malone performed the experiments. Dulce Mariscal and Gelsy Torres-Oviedo analyzed the data.

While subjects had similar aftereffects on the treadmill, we found that age strongly modulated the transfer of adapted movements from the treadmill to overground walking. Figure 4*A* shows the time courses of overground aftereffects. The youngest participants (red trace) appear to take a few steps overground before exhibiting large aftereffects in center of oscillation and step length asymmetry. However, this odd transfer pattern should be interpreted with caution since it was driven by two outliers. Figure 4*B* indicates the initial five strides recorded overground for individual subjects of all ages. Note the larger aftereffects in younger subjects for phase shift, which is reflected in the significant relation between age and OG_early_ values ( $r_{s}=0.61,p=2.3x10-5$). This indicates that the transfer of aftereffects across walking environments rapidly decreases as subjects’ age increases. This age effect was also observed in step symmetry ( $r_{S}=-0.61,p=2.9x10-5$) and center of oscillation ( $r_{S}=-0.33,p=0.03$). Interestingly, this effect was maintained after ∼200 steps of walking overground on step length asymmetry ( $r_{s}=-0.34,p=0.03$) and phase shift ( $r_{s}=0.40,p=0.01$). The relation between age and aftereffects is not observed during the late steps of center of oscillation ( $r_{s}=-0.24,p=0.12$; Fig. 4*C*). Thus, age modulated overground aftereffects and young children were still walking with asymmetric step lengths and asymmetric timing between the legs right before they returned to the treadmill.

### All children develop a motor memory specific to the treadmill

**Figure 5.**
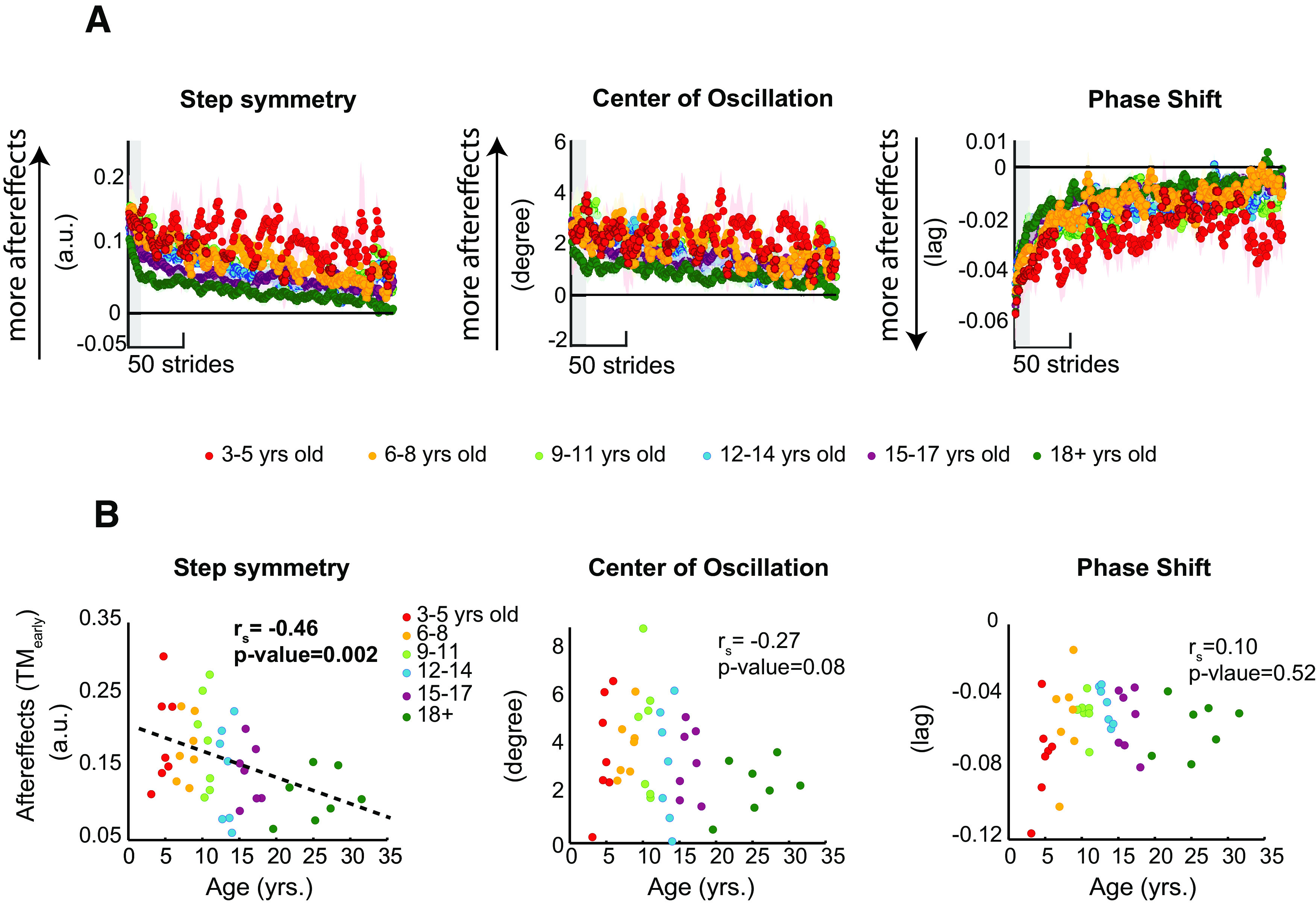
***A***, Time courses during treadmill walking postadaptation for two age groups. Every dot represents the group average of five strides and color shaded areas indicate SE. Gray area indicates the strides used for the regression analysis displayed in panels ***B***. ***B***, Correlations analyses showing the relation between age and aftereffects during the initial TM*_early_* steps of the postadaptation period on the treadmill in all kinematic parameters. Colors indicate the different age groups. Dots indicate individual subjects’ data. Age was a significant predictor of TM*_early_* in all step length asymmetry, such that younger subjects exhibited greater aftereffects overground than older children and young adults. Dotted line is shown as reference for a linear regression. Arbitrary units (a.u.).*Figure Contributions*: Gelsy Torres-Oviedo, Erin Vasudevan, and Laura Malone performed the experiments. Dulce Mariscal and Gelsy Torres-Oviedo analyzed the data.

We also observed an age effect on the washout of treadmill aftereffects by overground walking. Figure 5 shows the magnitude of remaining aftereffects on the treadmill following overground walking (TM_early_) versus Age for each parameter. Note that aftereffects are larger in younger individuals for step length asymmetry ( $r_{s}=-0.46,p=0.002$), but not in center of oscillation ( $r_{s}=-0.27,p=0.08$) or phase shift ( $r_{s}=0.1,p=0.52$). Larger persistent aftereffects were are observed during late postadaptation for the younger individuals (TM_late_; data not shown; step symmetry: $r_{S}=-0.48,p=0.001$; phase shift: $r_{s}=0.39,p=0.01$; center of oscillation: $r_{s}=-0.36,p=0.02$), indicating that younger adults have not fully de-adapted at the end of the postadaptation period.

### Young children that are intrinsically more variable transfer more

**Figure 6.**
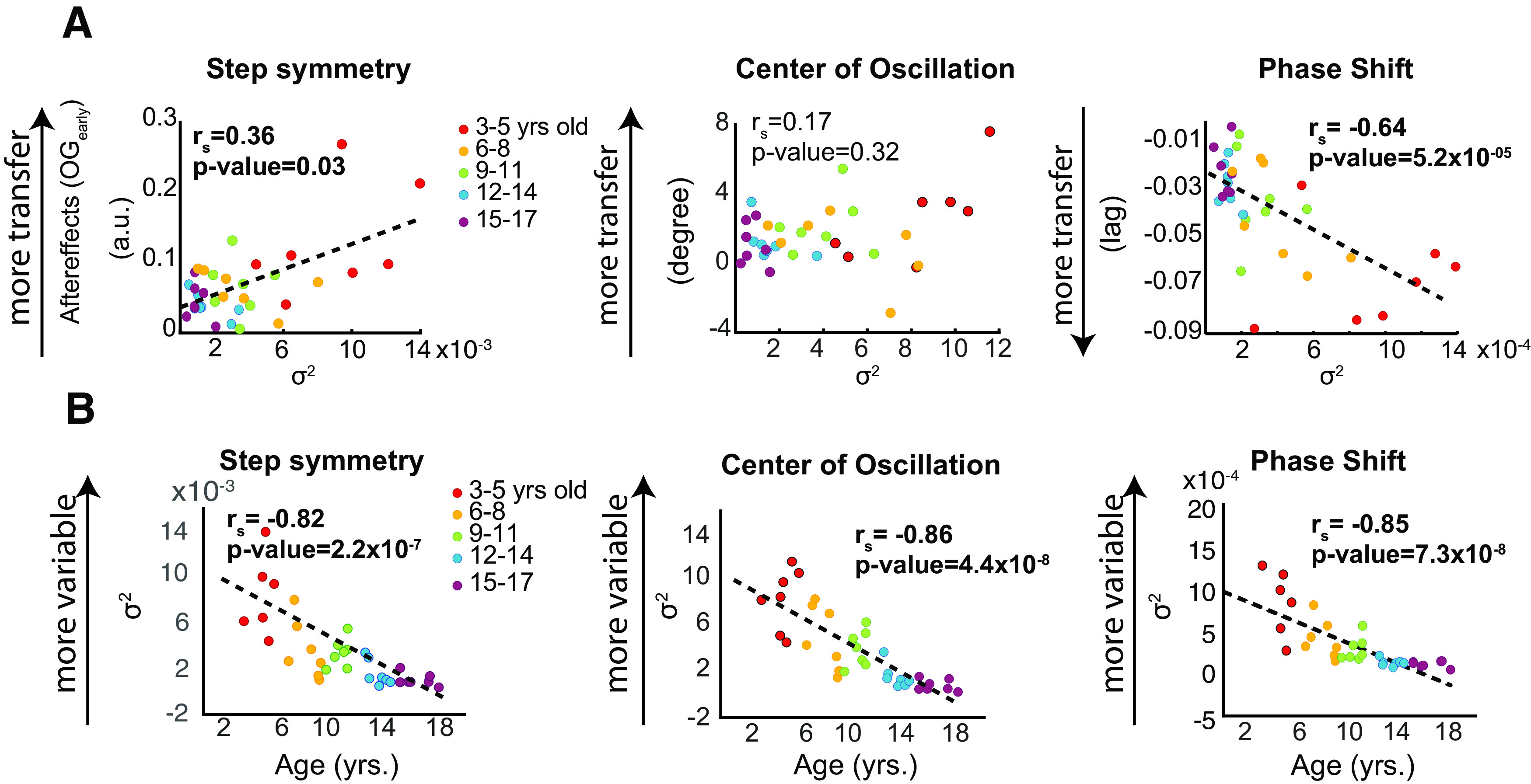
***A***, Scatter plots showing the relationship between variability in stepping and OG*_early_*. Colors indicate the different sensory conditions. Variability in behavior was a significant factor that predicted the transfer of adaptation effects to over ground walking. The magnitude of transfer was positively related to the subjects’ behavior variability: the more variable were subjects during adaptation, the more carry-over of aftereffects to over ground. ***B***, Scatter plots showing the correlation between age and baseline variability. There is a significant correlation between age and baseline variability in all gait parameters. These regressions show that children become less variable as in their gait pattern as they develop during childhood. Arbitrary units (a.u.).*Figure Contributions*: Gelsy Torres-Oviedo, Erin Vasudevan, and Laura Malone performed the experiments. Dulce Mariscal and Gelsy Torres-Oviedo analyzed the data.

We observed that subjects who had more variability in their walking tended to have larger aftereffects during overground walking. Figure 6 shows the observed OG_early_ values as a function of variance in step symmetry (left panel), center of oscillation (center panel), and phase shift (right panel) during baseline for each subject. Our regression analysis indicated that baseline variability was associated with the transfer of adaptation effects in step length symmetry $(r_{s}=037,p=0.03$) and phase shift ( $r_{s}-0.64,p=5.17x10-5$), but not center of oscillation ( r=0.17,p=0.32). This indicates that individual variability in each participant’s asymmetries could regulate the extent to which subjects transfer adapted movements across different environments. Note, however, that younger kids are also more variable than older kids. This is indicated by the association between subjects’ age and the variability associated with their step symmetry ( $r_{S}=-0.82,p=2.18x10\;-\;7$), center of oscillation ( $r_{s}=-0.86,p=4.42x10\;-\;8$), and phase shift ( $r_{s}=-0.85,p=7.25x10\;-\;8;$Fig. 6*B*). Thus, while motor variability during baseline and transfer appears to be positively related, this association might simply be the by-product of the relation between baseline variability and age.

## Discussion

Younger children generalize a newly learned walking pattern from the training environment (i.e., a split-belt treadmill) to an untrained environment (i.e., overground walkway) to a greater extent than older children and adults. We also found that differences in movement variability with age were correlated to differences in generalization, suggesting that movement variability in young subjects might play a role in the generalization of their learning across contexts. Finally, we found larger remaining aftereffects in younger than older children or adults when subjects returned to the treadmill after overground walking. While this was surprising because we would expect that the younger children who generalized more would also wash out more of the learned treadmill pattern, it could be explained by the fact that younger individuals are not fully de-adapted overground before they returned on the treadmill.

### Differences in generalization throughout childhood

Motor adaptation is a form of short-term learning that is induced by movement errors. Through trial-and-error practice, one learns to reduce errors and restore a more optimal movement pattern. One possible explanation for the generalization pattern that we observed following split-belt treadmill adaptation is related to error attribution during learning. Errors (i.e., perturbed movements) experienced in novel situations represent one contextual cue that might mediate the generalization of learning (Berniker and Kording, 2008; Torres-Oviedo and Bastian, 2012). It is thought that the motor system classifies errors as externally-generated or self-generated (Synofzik et al., 2006; Wilke et al., 2013). This classification affects both the extent of learning (Sober and Brainard, 2012; Kelly and Sober, 2014)and pattern of generalization (Berniker and Kording, 2008; Torres-Oviedo and Bastian, 2012). If errors are classified as externally-generated, the motor system associates learned movements to the environment, in this case, the treadmill. Consequently, the learning experience will impact untrained movements within the training environment, but the same motor behavior (e.g., walking) will remain unchanged when performed in other environments. Conversely, if errors are classified as self-generated, the motor system ties these corrections to the trained movement itself; thus, updated movements will generalize to other environments. This framework suggests that the generalization of learning is tightly coupled to the motor systems’ ability to recognize externally-generated versus self-generated errors accurately. Therefore, young children might generalize more than older kids or adults because error attribution matures as we develop.

Furthermore, young children’s variability might regulate their error attribution. Previous work suggested that people who are naturally more variable will generalize more because more of their errors during training will fall within the larger range of errors they normally experience (Torres-Oviedo and Bastian, 2012). Interestingly, we found greater variability in children’s walking behavior, which increases the possibility of attributing errors during split-belt walking to their own faulty movements and therefore generalizing more across different environments. It should also be noted that this idea is not unique to human behavior. For instance, it has been shown that young birds, compared with adult birds, compensate more for external perturbations to their songs when these perturbations fall within the natural range of produced sounds compared with when they fall out of this natural variability (Kelly and Sober, 2014). Here, we suggest that natural variability could also determine the generalization of motor learning across contexts.

The larger extent of generalization in young children could also be explained by ongoing development of neural substrates mediating the ability to disengage context-specific motor actions when switching from one environment to another. We observed younger children continued to perform the motor pattern for split-belt walking in the over ground context. Similar results are observed in adults older than 73 years old (Sombric et al., 2017) and people with stroke (Reisman et al., 2009), which are populations exhibiting either age-related structural decline (Fjell and Walhovd, 2010) or focal lesions to motor areas of the brain. It has been shown that cortical areas necessary for switching between motor programs are still developing during childhood (Wendelken et al., 2012). Therefore, young children might have difficulties disengaging newly learned locomotor patterns when the context changes because cerebral networks underlying action specificity continue to develop throughout youth.

Lastly, the immature executive function needed for task switching could also contribute to young children’s persistent motor actions across contexts. Multiple development studies have indicated that executive function, which includes the cognitive flexibility to switch actions, is developed during childhood (Zelazo, 2006; Prencipe et al., 2011) and through adolescence (Wilson et al., 2008). Children younger than five years old have difficulty in task switching: they perseverate in their actions based on rules applicable to a prior context (Zelazo, 2006). A possible explanation for this action perseveration is the inability to use contextual cues to switch actions in a proactive manner (Munakata et al., 2012). It is not until age 8 when the prefrontal cortex has reached the maturity needed for kids to change the course of ongoing actions (Chevalier et al., 2014). Thus, undeveloped cognitive functions in children such as cognitive flexibility might also contribute to children’s difficulties in switching locomotor patterns according to the walking condition.

### Similarities in learning and context specificity across age groups

While kids are slower learners than young adults (Vasudevan et al., 2011), participants from all ages were able to store new movement calibrations. This was indicated by the lack of age-effect on the treadmill aftereffects on the treadmill (before overground walking). Interestingly, younger participants adjusted their gait less during the split condition (ΔAdapt parameter), which could be explained by the fact that younger individuals adapt more slowly than older children or adults (Vasudevan et al., 2011). Despite the reduced gait changes during adaptation of younger children, they exhibited more aftereffects overground that were more resilient to washout from the overground experience. This suggests that a longer adaptation period on the treadmill for young children might lead to even greater aftereffects overground since gait changes on the treadmill results in larger aftereffects postadaptation (Sombric et al., 2019; Aucie et al., 2020).

While children from distinct age groups generalize their movements across environments differently, we do not observe differences in washout. Why is this the case? One possible explanation is that younger individuals are not washed out to the same level as older children and adults by the end of the overground period. This is consistent with previous studies showing that younger children take longer to washout the split-belt motor memory compared with older children or adults (Musselman et al., 2016). Thus, a longer washout period might be needed to have the same level of washout across groups. Another possibility might be that younger subjects have a reduced capacity to use contextual cues to transition between the tied and split motor pattern. In other words, older children and adults might learn from the split-to-tied transition during the catch condition to quickly switch to the tied motor pattern, whereas younger children have difficulties doing so. This is consistent with previous work showing that young children can use contextual cues to develop context-specific motor memories (Cox and Smitsman, 2006), but they have difficulties switching between them (Klossek and Dickinson, 2012).

### Clinical implications

Promising studies have shown that gait symmetry in adult and pediatric patients with a hemiparesis improves after split-belt walking (Reisman et al., 2013). Since the motor system changes during development, it is important to understand what motor learning mechanisms are available to children to optimize rehabilitation interventions in these patients. Here, we showed that children younger than 12 years are able to transfer more of the movement pattern that they acquired on the treadmill to natural walking compared with older children and adults. This suggests that rehabilitation effects will be conducted more to real life situations if children are trained in their early childhood. In sum, the generalization of motor learning is a mechanism available to children that can be beneficial for improving movements of pediatric populations beyond the clinical setting.

### Limitations

We note that arm swing was restricted differently between younger children and all other age groups, which could have led to distinct gait patterns. Namely young children (less than six y/o) walked with their arms extended holding a rail with both hands on the treadmill and overground, whereas older participants (6+ years old) walked with their arms crossed on the treadmill and overground. We had to introduce this methodological difference because young children were unable to maintain their arms crossed to prevent marker occlusion. We believe, however, that age-related differences in generalization are unlikely driven by this methodological distinction for two reasons. First, participants maintained the same arm position across contexts: young children held a rail on the treadmill and overground, whereas older participants crossed their arms on the treadmill and overground. Therefore, arm motion was not contextually different between age groups. Second, there have been other generalization studies in young adults during which arm swing is not constrained on the treadmill or overground, yet generalization patterns remain limited (Sombric et al., 2017; Mariscal et al., 2020) compared with the generalization observed in younger children

## Synthesis

Reviewing Editor: Ifat Levy, Yale University School of Medicine Decisions are customarily a result of the Reviewing Editor and the peer reviewers coming together and discussing their recommendations until a consensus is reached. When revisions are invited, a fact-based synthesis statement explaining their decision and outlining what is needed to prepare a revision will be listed below. The following reviewer(s) agreed to reveal their identity: Virginia Chu.

The generalization of motor learning from experienced situations to new ones is important for devising optimal rehabilitation programs. Little is known about how the motor system develops the ability to form generalizable versus context-specific motor memories. The authors found that younger children generalize locomotor patterns to a much greater extent than older children and adults. These results are significant because they demonstrate that the ability to form context-specific locomotor memories develops during childhood.

The study design is interesting and provides new insight on context-specific motor learning in children in the context of gait. The study is well designed and the manuscript is nicely written. Below are some comments that the authors need to address.

- The title seems to overstate the study’s conclusion. This study focused on motor learning specifically about gait, yet the title appears to generalize the study’s conclusion to all forms of motor learning.

- Subjects under 6 y/o were instructed to walk pushing a toy-shopping cart with adjustable rail height, and subjects over 6 y/o were asked to walk with their arms crossed. These adaptations may have changed the gait patterns, and could have created differences between younger and older children. Have the authors controlled for such potential effects? Also, why couldn’t younger children walk with their arms crossed to minimize age confounds? Please clarify if children pushing the toy cart did this with one or two hands, and provide information about the force used to push the trolley.

- Phase shift, center of oscillations, and step length symmetry were reported as change scores (difference from baseline) - were there any age-related differences in the absolute values in baseline?

- Figure 2B - several of the youngest subjects had a negative adaptation index in the step symmetry, what does that mean? Did they have more asymmetry at the end of adaptation vs the beginning? Similarly, there are a few of the youngest participants that had negative adaptation index in center of oscillation. It seems like the youngest group of participants is sometimes doing something that is qualitatively different from the rest of the participant group during the adaptation phase.

- Figure 3A is difficult to read as the shades overlap each other and much of the dots and shaded regions are covered.

- Figure 4A - the youngest participant group seemed to have more asymmetry and higher center of oscillation after the first 5 strides over ground. Is that driven by a few outliers? Or is it a representative pattern for very young kids, who take a few strides before transferring the split belt treadmill gait pattern to overground? That seems like an odd transfer pattern.

- Could the authors compare the OG_late and TM_early? If the children are forming different motor patterns for treadmill vs overground walking, there should be a significant difference between OG_late and TM_early, as the aftereffects would jump back to higher levels as though there was no washout.

- The clinical implications section discussed the rehab effect of transferring more of the movement pattern from treadmill to natural walking. However, all previous research, as well as this study, showed that the aftereffects eventually washout. If there is no long-term lasting effects, how is that useful for rehab benefits?

- Please add a table with subject characteristics and the number of analyzed strides and range of speeds.

- Statistical analysis: were the data normally distributed? Is the use of regression analysis justified here?

## Author Response

Dear Editor,

Hereby we re-submit our manuscript ’Context-specificity of locomotor learning is developed during childhood’ to eNeuro. We kindly request this manuscript to be considered for publication as New Research within the Topic of Cognition and Behavior in this journal.

We truly appreciate the reviewers’ thorough comments on our original manuscript and your careful assessment of these documents. We have made a concerted effort to address the reviewers’ feedback. We strongly believe that the revised document is greatly improved and we sincerely appreciate the opportunity to undergo the reviewing process to be potentially accepted at eNeuro.

Below we provide a response to the reviewers providing a line-by-line response (marked in italic bold letters) to the reviewers’ concerns.

We remain very interested to contribute to this journal and think our work will be well regarded by the audience of eNeuro. We look forward to the reviewers revision of our updated document.

Synthesis of Reviews:

Computational Neuroscience Model Code Accessibility Comments for Author (Required): N/A

Significance Statement Comments for Author (Required):

appropriate

Comments on the Visual Abstract for Author (Required): N/A

Synthesis Statement for Author (Required):

The generalization of motor learning from experienced situations to new ones is important for devising optimal rehabilitation programs. Little is known about how the motor system develops the ability to form generalizable versus context-specific motor memories. The authors found that younger children generalize locomotor patterns to a much greater extent than older children and adults. These results are significant because they demonstrate that the ability to form context-specific locomotor memories develops during childhood.

The study design is interesting and provides new insight on context-specific motor learning in children in the context of gait. The study is well designed, and the manuscript is nicely written. Below are some comments that the authors need to address.

1. The title seems to overstate the study’s conclusion. This study focused on motor learning specifically about gait, yet the title appears to generalize the study’s conclusion to all forms of motor learning.

Response to reviewers:

We appreciate the reviewer’s suggestion. We have changed the title of the manuscript to specify that this study is related to gait.

The revised title reads:

"Context-specificity of locomotor learning is developed during childhood"

2. Subjects under 6 y/o were instructed to walk pushing a toy-shopping cart with adjustable rail height, and subjects over 6 y/o were asked to walk with their arms crossed. These adaptations may have changed the gait patterns and could have created differences between younger and older children. Have the authors controlled for such potential effects? Also, why couldn’t younger children walk with their arms crossed to minimize age confounds? Please clarify if children pushing the toy cart did this with one or two hands and provide information about the force used to push the trolley.

Response to reviewers:

We acknowledge that the arm motion has an impact on gait patterns. Unfortunately, young children

were unable to consistently maintain their arms crossed as they walked. Thus, we had to use a light (7- pounds) toy cart during overground walking and a handrail during treadmill walking to prevent them from occluding the markers on their hips and pelvis. Also, we did not record forces on either the cart or the treadmill. We have updated our discussion to point out these study limitations.

We, however, think that age-related differences in generalization are unlikely driven by the methodological distinction in arm restrictions between groups. Note that participants maintained the same arm positions across contexts. In other words, young children (<6 y/o) walked with their arms extended holding a rail with both hands on the treadmill and overground, whereas older participants (6+ years old) walked with their arms crossed on the treadmill and overground. Moreover, generalization patterns in which arm swing is not restricted (Sombric et al 2017, Mariscal et al. 2020) do not reach those observed in younger children.

The updated text in the methods reads:

"To avoid occlusion of the hip and pelvis markers during overground and treadmill walking due to arm- swing, participants walked with their arms crossed on the treadmill and overground, except for young children (< 6 years old) who were unable to consistently maintain their arms crossed while walking. Thus, young children were instructed to walk holding a rail with both hands on the treadmill and overground while pushing a light (7 lb) toy-shopping cart with adjustable rail height.”

The updated text in the discussion reads:

"We note that arm swing was restricted differently between younger children and all other age groups, which could have led to distinct gait patterns. Namely young children (<6 y/o) walked with their arms extended holding a rail with both hands on the treadmill and overground, whereas older participants (6+ years old) walked with their arms crossed on the treadmill and overground. We had to introduce this methodological difference because young children were unable to maintain their arms crossed to prevent marker occlusion. We believe, however, that age-related differences in generalization are unlikely driven by this methodological distinction for two reasons. First, participants maintained the same arm position across contexts: young children held a rail on the treadmill and overground, whereas older participants crossed their arms on the treadmill and overground. Therefore, arm motion was not contextually different between age groups. Second, there have been other generalization studies in young adults during which arm swing is not constrained on the treadmill or overground, yet

generalization patterns remain limited (Sombric et al 2017, Mariscal et al. 2020) compared to the generalization observed in younger children”.

3. Phase shift, center of oscillations, and step length symmetry were reported as change scores (difference from baseline) - were there any age-related differences in the absolute values in baseline?

Response to reviewers:

Thanks to the reviewers for their comments.

No, there are no age-related differences on participants baseline behavior (SLA, 2(5)=3.76,p=0.59;

center of oscillation 2(5)=1.12,p=0.95; phase shift 2(5)=7.85,p=0.16). However, due to their participants’ baseline behavior variability, we decided to run our analysis in unbiased data.

4. Figure 2B - several of the youngest subjects had a negative adaptation index in the step symmetry, what does that mean? Did they have more asymmetry at the end of adaptation vs the beginning? Similarly, there are a few of the youngest participants that had negative adaptation index in center of oscillation. It seems like the youngest group of participants is sometimes doing something that is qualitatively different from the rest of the participant group during the adaptation phase.

Response to reviewers:

Per the reviewer’s question, we inspected individual time courses for young children (red dots in

manuscript). See attached figure illustrating the time courses for all the participants (Panel A) and for the participants with negative “adaptation index” (Panel B). The gray line in Panel A and B indicates the averaged time courses.

Notice that none of the subjects with negative “adaptation indices” become more asymmetric during the adaptation period. However, all participants are very variable. Thus, it is difficult to capture the initial values for these two parameters. This high variability leads to apparent negative “adaptation” indices for some participants. We do not observe similar level of variability in phase shift.

We have added a sentence to the manuscript commenting that the negative values for spatial parameters are due to high stepping variability in those children, rather than an increase in asymmetric stepping.

The updated text reads:

"Of note, we observed negative Adapt indices in a couple of youngest participants because their large step-to-step variability in spatial parameters (step length asymmetry and center of oscillation) made it difficult to quantify their initial adaptation values. Thus, these negative indices represent large step-to- step variability in these youngest subjects, rather than an increment in asymmetric stepping during the adaptation period. “

5. Figure 3A is difficult to read as the shades overlap each other and much of the dots and shaded regions are covered.

Response to reviewers:

Thanks to the reviewers for their comment, we updated the figure to address this concern. Update figure

6. Figure 4A - the youngest participant group seemed to have more asymmetry and higher center of oscillation after the first 5 strides over ground. Is that driven by a few outliers? Or is it a representative pattern for very young kids, who take a few strides before transferring the split belt treadmill gait pattern to overground? That seems like an odd transfer pattern.

Response to reviewers:

The reviewer is correct that the odd dynamics in center of oscillation values during overground walking is driven by an outlier (yellow trace). We made a note of it in the updated results.

The updated text reads:

"The youngest participants (red trace) appear to take a few steps overground before exhibiting large aftereffects in center of oscillation and step length asymmetry. However, this odd transfer pattern should be interpreted with caution since it was driven by two outliers.”

7. Could the authors compare the OG_late and TM_early? If the children are forming different motor patterns for treadmill vs overground walking, there should be a significant difference between OG_late and TM_early, as the aftereffects would jump back to higher levels as though there was no washout.

Response to reviewers:

Per the reviewer’s request we performed a paired analysis confirming that participants developed a

context-specific motor memory during split-belt walking. More specifically, we computed a difference between aftereffects on the treadmill and overground (i.e., TM_early- OG_late) to test the hypothesis that “aftereffects would jump back to higher levels as though there was no washout”. We indeed found that TM_early-OG_late is significantly different from zero using a Wilcoxon signed-rank test (Z=-5.65, p=1.64x10-8), indicating that the initial aftereffects on the treadmill (TM_early) are larger than the aftereffects overground (during late post-adaptation).

8. The clinical implications section discussed the rehab effect of transferring more of the movement pattern from treadmill to natural walking. However, all previous research, as well as this study, showed that the aftereffects eventually washout. If there is no long-term lasting effects, how is that useful for rehab benefits?

Response to reviewers:

The reviewer is correct that the effects washout out in single session studies, such as our study. However, there is evidence that multiple exposure to split-belt walking can change the step length asymmetry for children (Choi et al. 2009) and adults with hemiparetic gait (Reisman et al. 2013, Lewek et al. 2018, Betshart et al., 2018). We include a clinical section to our discussion because our results indicate an age- related dependency on the generalization of motor patterns beyond the treadmill context.

9. Please add a table with subject characteristics and the number of analyzed strides and range of speeds. (Age, sex, height, leg length, walking speed, range of speeds, analyzed strides)

Response to reviewers:

Per reviewers’ request, we updated the table on our manuscript, which now includes the sex distribution and average slow and fast speeds per group. We did not add to the table the participants leg length since it is the same as the value as the slow speed that they experienced. This is indicated in the methods section. We could not include the participants’ height because we, unfortunately, did not collect this information. Lastly, the number of analyzed steps were not included in the table because these depend on the epochs of interest (as stated in the methods). For example, in all participants 40 steps were included in the “late” epochs, whereas 5 steps were included in the computation of the “early” epochs.

10. Statistical analysis: were the data normally distributed? Is the use of regression analysis justified here?

Response to reviewers:

We sincerely appreciate the reviewer’s suggestion. After testing for normality, we found that our data sample and the residuals of our regression do not satisfy the normality assumption. Thus, we updated our methods and results with a non-parametric Spearman correlation. We found that the conclusions for the study have not changed.
